# Synthesis and characterization of novel polyimides derived from 4,4’-bis(5-amino-2-pyridinoxy)benzophenone: effect of pyridine and ketone units in the main

**DOI:** 10.1080/15685551.2016.1231036

**Published:** 2016-09-28

**Authors:** Chunbo Wang, Xiaogang Zhao, Dongbo Tian, Daming Wang, Chunhai Chen, Hongwei Zhou

**Affiliations:** ^a^ Alan G. MacDiarmid Institute, Jilin University, Changchun, PR China

**Keywords:** Polyimide, pyridine, heterocyclic diamine, nucleophilic substitution reaction

## Abstract

A diamine monomer, 4,4’-bis(5-amino-2-pyridinoxy)benzophenone, was designed and synthesized, and used to react with commercially different kinds of aromatic dianhydrides to prepare a series of polyimides containing pyridine and ketone units via the classical two-step procedure. Glass transition temperatures (*T*_g_) of the resultant polyimides PI-(1–5) derived from 4,4’-bis(5-amino-2-pyridinoxy) benzophenone with various dianhydrides ranged from 201 to 310 °C measured by differential scanning calorimetry. The temperatures for 5%wt loss of the resultant polyimides in nitrogen atmosphere obtained from the thermogravimetric analysis curves fell in the range of 472–501 °C. The temperatures for 10%wt loss of the resultant polyimides in nitrogen atmosphere fell in the range of 491–537 °C. Meanwhile, the char yields at 800 °C were in the range of 55.3–60.8%. Moreover, the moisture absorption of polyimide films was measured in the range of 0.37–2.09%. The thin films showed outstanding mechanical properties with tensile strengths of 103–145 MPa, an elongation at break of 12.9–15.2%, and a tensile modulus of 1.20–1.88 Gpa, respectively. The coefficients of thermal expansion of the resultant polyimides were obtained among 26–62 ppm °C^−1^. To sum up, this series of polyimides had a good combination of properties applied for high-performance materials and showed promising potential applications in the fields of optoelectronic devices.

## 1. Introduction

Advanced polyimides have been extensively used in the fields of the aerospace, microelectronics, functional membranes, advanced composite for their exceptional thermal stability, outstanding mechanical properties, low dielectric constant and great chemical resistance.[1–6] However, with the rapid progress of high-tech fields, higher requirements are simultaneously put forward to the polymer material performances. In recent years, considerable attention has been devoted to synthesis novel diamines or dianhydrides possessing a special structure, such as fluoride structure, symmetrical and unsymmetrical structure, ketone-based structure, heterocyclic units, etc.[7–11] Ketone-based compounds have been receiving more attention as a monomer for the synthesis of polyimides due to possess the desirable properties.

In the classical polyimides, introducing ketone unit into the molecular chain can effectively change some properties of the polymer, resulting in high thermal stability, mechanical properties, but low transparency. This disadvantage of the polyimide hinders its application in the field of optoelectronic devices. It is an effective method to modify optical property of polyimide by incorporating bulkily fluorinated groups, aliphatic segments, and heterocyclic units into polymer backbone.[10,12–14] The incorporation of aromatic heterocyclic structure into the backbone of the polymers results in imparting certain properties to the polymer while not deteriorating original outstanding properties. Pyridine belongs to heterocyclic units, and nitrogen atoms with a free electron give an opportunity for protonation to modify optical property.[6,15] If the ortho and para positions have better leaving group (such as halogen, nitro) in pyridine moiety, it is easier to take place nucleophilic substitution reaction. It is an easy way that incorporates pyridine ring into polymer backbones. So, the presence of ketone and pyridine groups lead to keep rigidity and improve optical property, these polyimides have potential applications in the fields of optoelectronic devices.

In this article, a new monomer was designed by incorporating two special structure units simultaneously into polyimide backbones while the desirable properties are of particular interest. A diamine containing pyridine and ketone units, 4,4’-bis(5-amino-2-pyridinoxy) benzophenone (BADBP), was synthesized and the corresponding polyimides were prepared based on BADBP with five commercial dianhydrides by the classical two-step polymerization procedure. The influences of incorporating two special units, pyridine and ketone, on the thermal stability, mechanical property, optical transparency, solubility and so on were systematically investigated.

## 2. Experimental

### 2.1. Starting materials

The dianhydrides, including 4,4’-(Hexafluoroisopropylidene) diphthalic anhydride (6FDA), 3,3’,4,4’-oxydiphthalic anhydride (ODPA), 22-bis[4-(3,4dicarboxyphenoxy) phenyl] propane dianhydride (BPADA), 3,3’,4,4’-benzophenonetetracarboxylic dianhydride (BTDA) and pyromellitic dianhydride (PMDA), were supplied by Sinopharm Chemical Reagent Beijing Co. Ltd and were baked at 110 °C in vacuo overnight prior to use. Potassium carbonate (K_2_CO_3_) was supplied by Acros and was dried in vacuum at 130 °C for 10 h prior to use. N,N-dimethylacetamide (DMAc) and N,N-dimethylformamide (DMF) were purified by distillation under reduced pressure over calcium hydride and stored over 4 Å Molecular sieves before use. 2-chloro-5-nitropyridine, 4,4’-dihydroxybenzophenone, and SnCl_2_·2H_2_O were purchased from Acros without further purification.

### 2.2. Measurements

#### 2.2.1. Elemental analysis

Elemental analysis was run on a Vario EL cube CHN recorder elemental analysis instrument.

#### 2.2.2. Inherent viscosities (*ɳ*_inh_)

*ɳ*_inh_ of all the PAAs (Polyamide Acids) in DMAc solvent were measured with a content of 0.5 g/dL using an Ubbelohde viscometer at 25 °C.

#### 2.2.3. Fourier transform infrared spectrometer (FT-IR)

FT-IR was recorded on a Bruker Vector22 spectrometer using KBr (potassium bromide) pellets or about 10um thick polymer films.

#### 2.2.4. ^1^H NMR

^1^H NMR spectra of the polyimides were recorded on a BRUKER 300 MHz instrument using DMSO-d_6_ as solvent.

#### 2.2.5. Differential scanning calorimetric analysis (DSC)

DSC was performed on a TA Instruments (TA Q100) with a heating rate of 10 °C/min in a nitrogen flowing condition at 50 ml/min, and the results determined by the second heating cycle.

#### 2.2.6. Thermogravimetric analysis (TGA)

Thermogravimetric data was obtained on TA 2050 under the nitrogen flowing condition at a heating rate of 10 °C/min.

#### 2.2.7. Dynamic mechanical analysis (DMA)

DMA was performed on TA instrument (TA Q800), and *T*_g_ decided by the peak temperature of loss modulus (*E*″).

#### 2.2.8. Thermomechanical analysis (TMA)

TMA was performed on METTLER instrument (TMA/SDTA841), and the coefficient of thermal expansion (CTE) decided by the temperature range of 50–150 °C.

#### 2.2.9. Ultraviolet–visible (UV–vis) spectra

UV–vis spectra were measured with a Shimadzu UV–vis 2501 spectrometer in transmittance mode using the thin films as sample at room temperature.

#### 2.2.10. Mechanical analysis

Tensile property of the polymer films was measured with a Shimadzu AG-I universal testing apparatus with crosshead speed of 5 mm/min at room temperature,and the thin film specimen sizes were at 25–30um thick, 3 mm wide and 4 cm long.

#### 2.2.11. Water uptakes

Water uptakes (WU) of the films were calculated by the following equation: WU = [(*W*_wet_ − *W*_dry_)/*W*_dry_] × 100%; where *W*_wet_ refers to the weight of PI film samples after immersion in deionized water at room temperature for 24 h, and *W*_dry_ is the initial weight of them.

### 2.3. Synthesis of the monomers

#### 2.3.1. 4,4’-bis(5-nitro-2-pyridinoxy)benzophenone (BNDBP)

Under nitrogen protection, 4,4’-dihydroxybenzophenone (6.43 g, 30 mmol), 2-chloro-5-nitropyridine (10.47 g, 66 mmol), potassium carbonate (9.12 g, 66 mmol) and 75 ml of dried DMF were charged into a 250 ml reaction flask with a mechanical stirrer and reflux condenser. After 30 min of stirring at room temperature, the reaction mixture was continuously reacted at 80 °C for 6 h. After cooling to room temperature, the mixture was poured into 250 ml of deionized water. The solid power was collected by filtration and washed thoroughly with water. The crude product was then dried in vacuum at 110 °C overnight. The yellow solid was recrystallized from DMF/water, and the yield of the product (BNDBP) was 15.05 g (85%). Melting point: 208 °C. FT-IR (KBr): 1649, 1598, 1576, 1518, 1463, 1391, 1350, 1280, 1261, 1199, 1162, 1115. ^1^H NMR: 8.73–8.38 (m, 2H), 9.29–8.93 (m, 2H), 7.95 (d, 4H), 7.29 (dd, 4H), 7.15 (s, 2H). Elemental Analysis Calcd: C, 60.27%; H, 3.08%; N, 12.22%. Found: C, 60.31%; H, 3.15%; N, 12.39%.

#### 2.3.2. 4,4’-bis(5-amino-2-pyridinoxy)benzophenone (BADBP)

Under nitrogen protection, 10.0 g (21.8 mmol) of BNDBP, and 49.2 g (218.0 mmol) of SnCl_2_·2H_2_O were charged into a reaction flask, meanwhile 47 ml of concentrated hydrochloric acid was added slowly. After addition of hydrochloric acid was finished, the mixture was refluxed for 7 h. The reaction mixture was poured into 400 ml of distiller water when cooling to room temperature. The mixing solution was basified with 40%NaOH solution to form a precipitate. The precipitate was filtrated off, washed with water, and recrystallized from ethanol to get a white product.[16] After dried under vacuum at 80 °C for overnight, 6.3 g of BADBP was obtained (63%). Melting point: 171 °C. FT-IR (KBr): 3443, 3346, 3209, 2361, 1640, 1595, 1478, 1414, 1309, 1277, 1239, 1157. ^1^H NMR: 7.74 (d, 4H), 7.63 (s, 2H), 7.12 (s, 2H), 7.06 (d, 4H), 6.96–6.82 (m, 2H), 5.27 (s, 4H). Elemental Analysis Calcd: C, 69.34%; H, 4.55%; N, 14.06%. Found: C, 68.97%; H, 4.54%; N, 13.58%.

### 2.4. Synthesis of the polyimides

#### 2.4.1. Poly(amic acid) synthesis

**Scheme 1.**
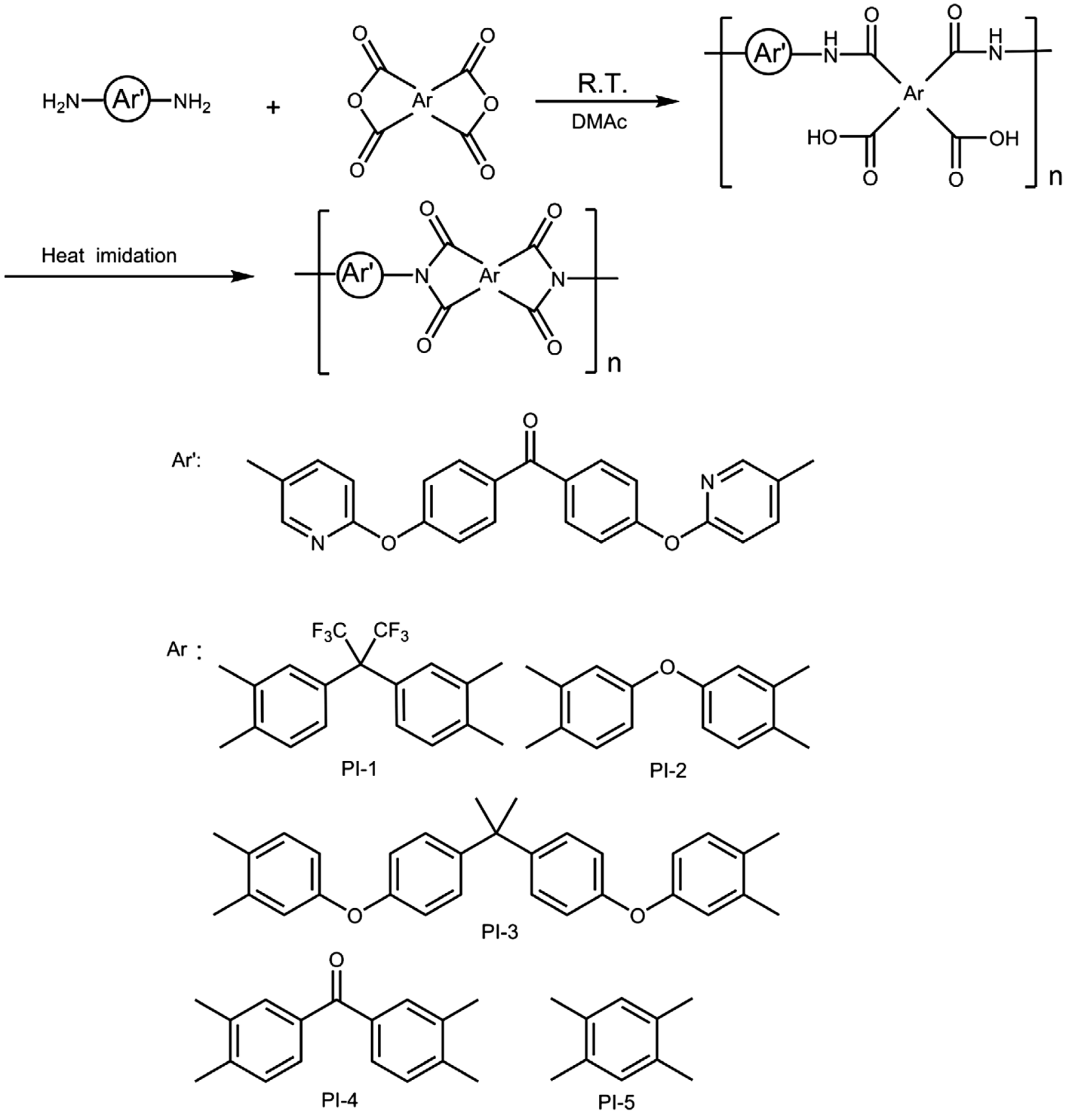
Synthesis route of the polyimides.

As shown in Scheme 1, BADBP was used to react with commercially five aromatic dianhydride (6FDA, PMDA, BTDA, ODPA and BPADA, respectively) to prepare a series of heterocyclic polyimides (PI-1–PI-5) via a typical two-step polymerization procedure. In case of PI-1 derived from BADBP with 6FDA, PAA was obtained as following procedure: 0.56 g 6FDA (1.25 mmol) was slowly added to the solution of BADBP (0.50 g, 1.25 mmol) in 6.0 ml DMAc at a solid content 15%wt. Then, the reaction mixture was stirred at room temperature for 12 h under N_2_ protection to yield the viscous PAA solution.

Similarly, the other PAAs were obtained by the above-mentioned procedures.

#### 2.4.2. Preparation of polyimide films

Polyimide film was prepared by the casting of 15%PAA onto the clean glass plate. The glass plate was placed in an oven and allowed to elevate temperatures (60 °C/2 h; 80 °C/2 h; 100 °C/2 h; 120 °C/1 h; 150 °C/1 h) to remove the solvents in air. Then the heating imidization procedure (200 °C/0.5 h, 250 °C/0.5 h, 300 °C/1 h) was performed in imine furnace under vacuum. The freestanding PI film was obtained by soaking in water to release from the glass plate. [17]

Similarly, PI-2–PI-5 films were obtained by the above-mentioned procedures.

## 3. Results and discussion

### 3.1. Synthesis of diamine monomer

**Figure 1.**
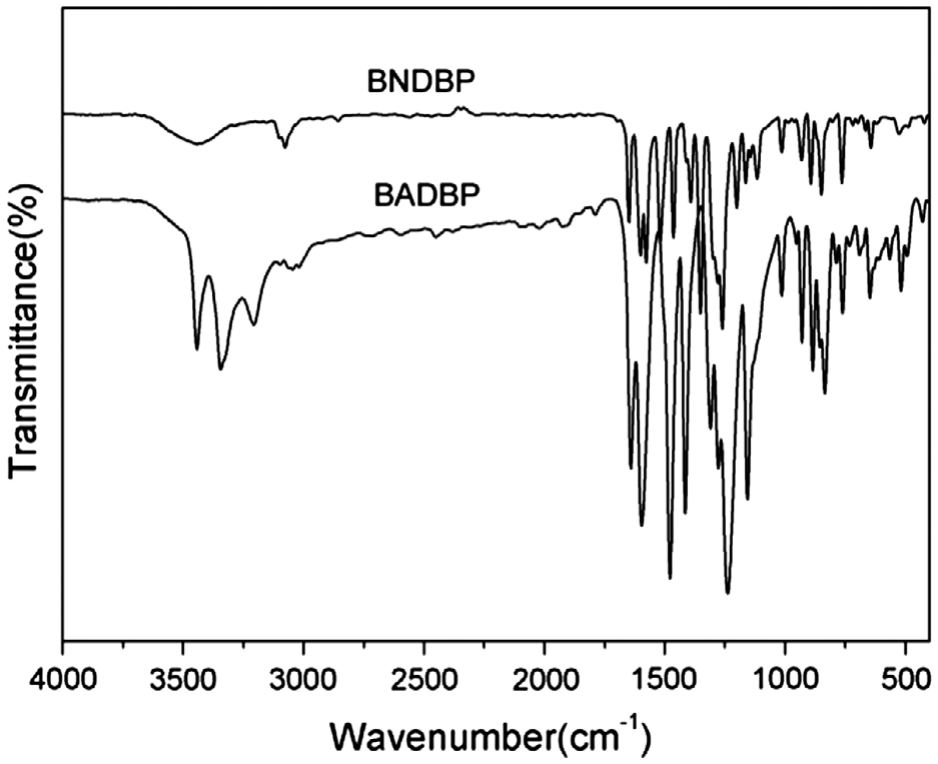
FT-IR spectra of dinitro intermediate and diamine monomer.

**Figure 2.**
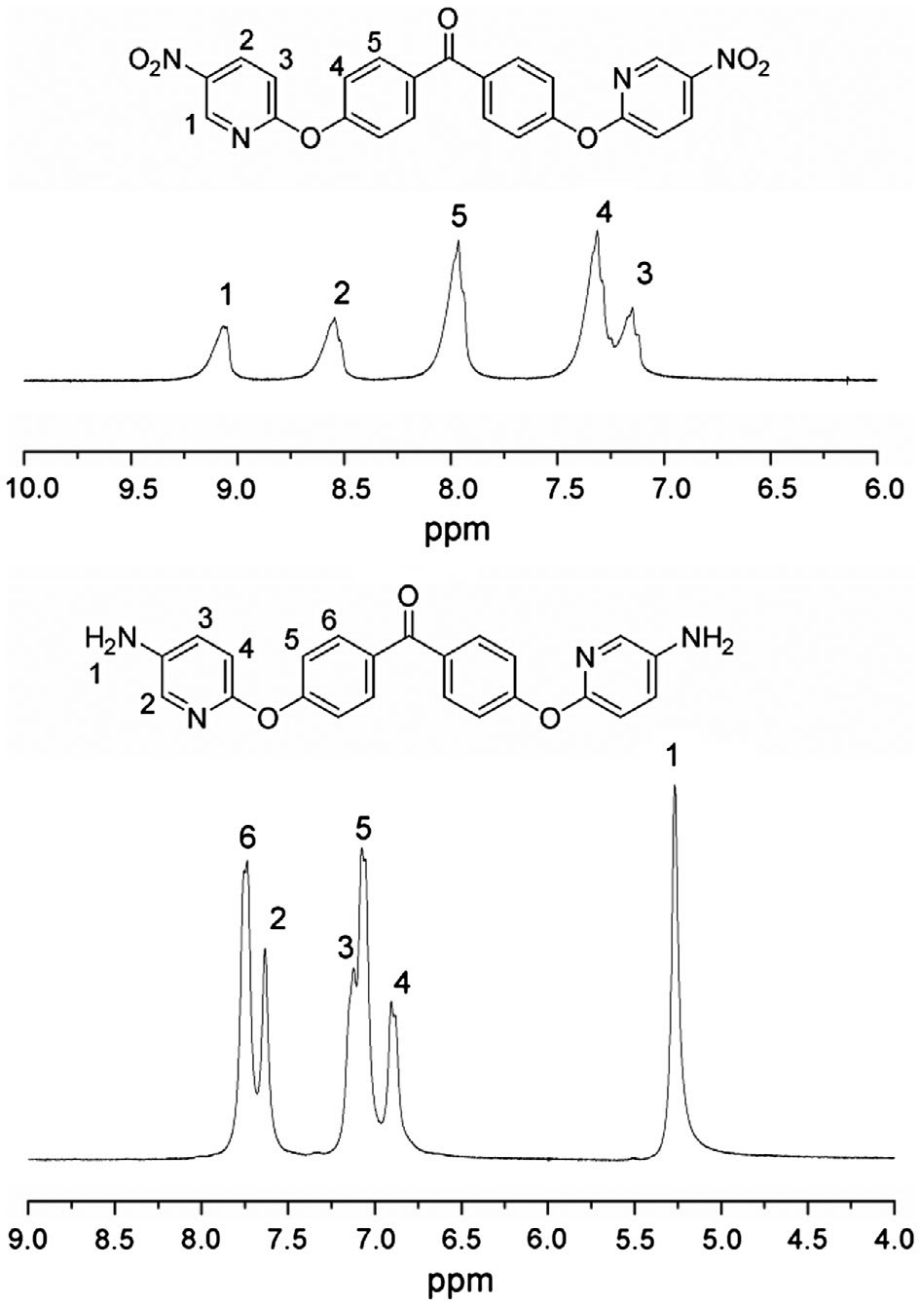
^1^H NMR spectra of BNDBP and BADBP monomers.

**Scheme 2.**
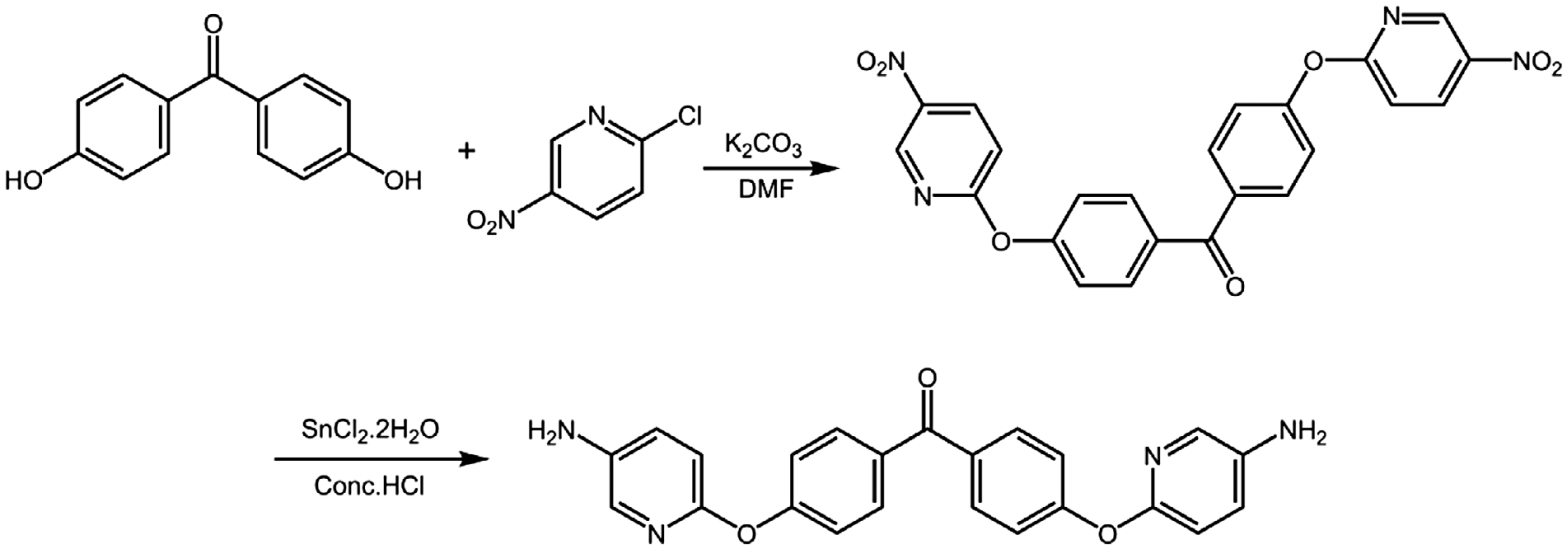
Synthesis the monomer containing pyridine and ketone units.

The synthesis of the novel diamine containing heterocyclic pyridine and ketone groups, 4,4’-bis(5-amino-2-pyridinoxy)benzophenone, was shown in Scheme 2. The nitro compound was synthesized with nucleophilic substitution reaction starting from 4,4’-dihydroxybenzophenone and 2-chloro-5-nitropyridine in the presence of potassium carbonate in DMF by stirring of the mixture at 80 °C for 6 h. The chlorine atom activated by the para nitro-group and ortho nitrogen-atom was readily carried out the chlorine-displacement reaction of 2-chloro-5-nitropyridine by the phenoxide anion. The diamine was readily obtained in a good yield by the catalytic reduction of the intermediate dinitro compound with SnCl_2_·2H_2_O and concentrated hydrochloric acid at refluxing temperature. The structures of BADBP and BNDBP were confirmed by elemental analysis, FT-IR spectra, and ^1^H NMR spectroscopy. Figure 1 shows the FT-IR spectra of BNDBP and BADBP, the nitro group of BNDBP gave two characteristic bands at 1518 and 1391 cm^−1^ (–NO_2_ asymmetric and symmetric stretching), respectively. After the reduction, the characteristic absorptions of the nitro group disappeared, and N–H absorption peaks of the amino group in the region of 3210–3450 cm^−1^ were detected. As shown in Figure 2, ^1^H NMR spectrum of the diamine monomer (BADBP) illustrates that the nitro groups in BNDBP were completely reduced, and the signal of amino groups appeared at around *δ*5.26 as a singlet. All the spectroscopic data were in agreement with the expected structures.

### 3.2. Synthesis and characterization of polyimides

**Figure 3.**
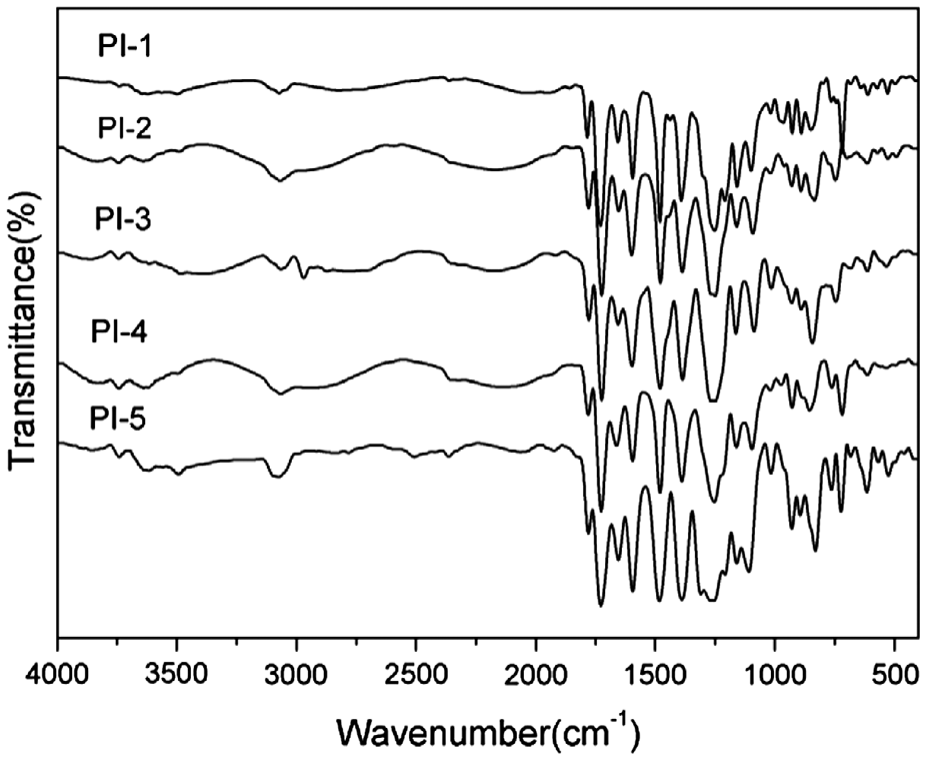
FT-IR spectras of the polyimide films.

The diamine, BADBP, was used to react with commercially different aromatic dianhydrides (6FDA, PMDA, BTDA, ODPA and BPADA, respectively) to yield a series of heterocyclic polyimides (PI-1–PI-5) via a typical two-step polymerization method. PAA was prepared by reacting equimolar amounts of diamine monomer with commercially aromatic dianhydride at a solid content of 15wt%, and stirred for 12 h at room temperature under N_2_ atmosphere. The polyimide film was prepared by the casting of 15%PAA onto the clean glass plate. The glass plate was placed in an oven and allowed to elevate temperatures (60 °C/2 h; 80 °C/2 h; 100 °C/2 h; 120 °C/1 h; 150 °C/1 h) to remove the solvents in air. The heating imidization procedure (200 °C/0.5 h, 250 °C/0.5 h, 300 °C/1 h) was performed in imine furnace under vacuum. As shown in Table 2, the inherent viscosities of the PAAs were in the range of 0.34–0.57 dL/g measured at a content of 0.5 g/dL in DMAc at 25 °C. In FT-IR spectra of PIs, shown in Figure 3, the characteristic imide absorption bands (PI-1–PI-5) were detected for 1785, 1779, 1778, 1780, 1779 cm^−1^ (asymmetrical C=O stretching), 1728, 1724, 1724, 1726, 1727 cm^−1^ (symmetrical C=O stretching), 1391, 1386, 1385, 1388, 1388 cm^−1^ (C–N stretching), and N–H absorption peak among 3210–3450 cm^−1^ disappeared because the PAAs had been full imidization. As shown in Table 1, these found values were in agreement with the calculated ones of C, H, and N in elemental analyses of the approved molecular formulas, and the yield of polyimides was in the range of 95–97%.

**Table 1.** Elemental analysis and yield of the polyimides.

Code	Formula of repeating unit	Elemental analysis (%)								Yield (%)
				C		H		N		
PI-1	C_42_H_20_F_6_N_4_O_7_	Calcd	found	62.54	61.59	2.50	2.79	6.95	6.78	97
PI-2	C_39_H_20_N_4_O_8_	Calcd	found	69.64	68.76	3.00	3.11	8.33	8.35	97
PI-3	C_54_H_34_N_4_O_9_	Calcd	found	73.46	72.81	3.88	3.93	6.35	6.72	96
PI-4	C_40_H_20_N_4_O_8_	Calcd	found	70.18	68.64	2.94	3.22	8.18	8.77	96
PI-5	C_33_H_16_N_4_O_7_	Calcd	found	68.28	67.88	2.78	3.04	9.65	10.27	95

### 3.3. Thermal properties of polyimides

**Figure 4.**
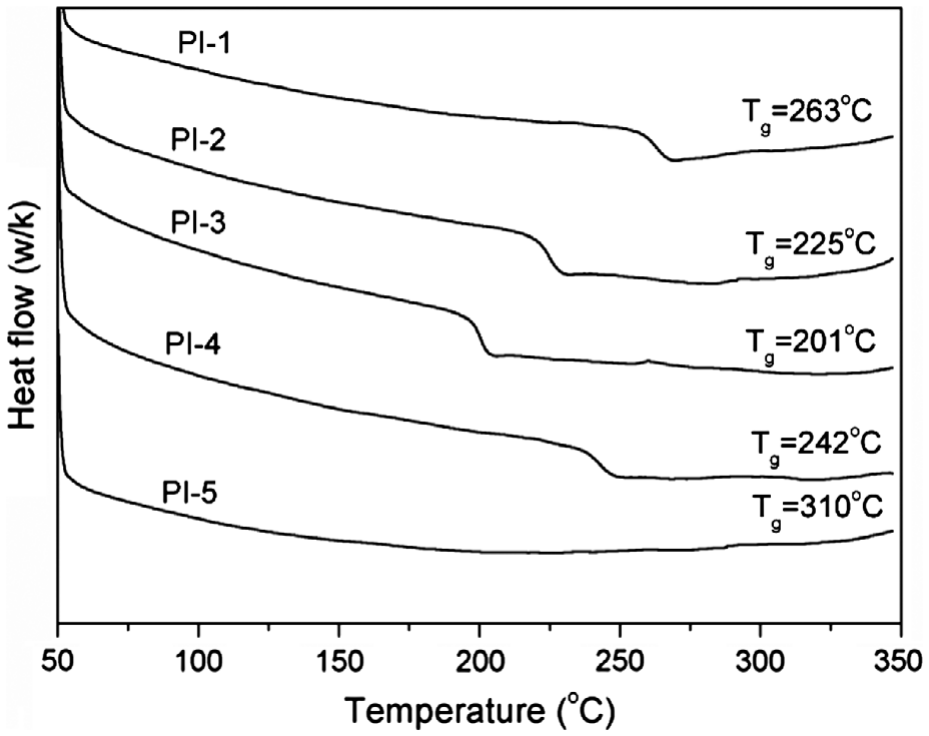
DSC curves of the polyimide films.

As shown in Table 2, thermal properties of the polyimides were detected by DSC, TGA, DMA and TMA. In DMA curves, *T*_g_ was decided by the peak temperature of loss modulus (*E*″). Glass transition temperatures (*T*_g_) of the polyimides (PI-1–PI-5) derived from BADBP with various dianhydrides in the range of 201–310 °C obtained by DSC in Figure 4 and 147–291 °C obtained by DMA in Figure 6, respectively. All the polyimides exhibited outstanding thermal property. It is probably attributed to incorporate ketone and pyridine units in polymer backbones. Generally, *T*_g_ values of polymers are determined by rigidity of the polymer backbones. The rigidity of dianhydrides is listed as follow: PMDA > BTDA > 6FDA > ODPA > BPADA. PI-5 derived from PMDA possessed the highest *T*_g_ due to its rigidity polymer chain structure, and PI-3 derived from BPADA possessed the lowest *T*_g_ due to its flexible polymer chain structure. However, PI-1 derived from 6FDA showed higher *T*_g_ value than that of PI-4 derived from BTDA. This exception might be attributed to inhibit the free rotation of the polymer segments due to the existence of –CF_3_ group in the polyimide backbone.[18]

**Table 2.** Thermal properties of the PI films.

Code	*ɳ*_inh_ of PAA (dL/g)^a^	*T*_g_ (°C)		*T*5% (°C)^c^	*T*10% (°C)^c^	Rw (%)^d^	CTE^e^
		DSC^a^	DMA^b^				
PI-1	0.37	263	248	501	537	57.6	60
PI-2	0.34	225	206	487	518	58.5	32
PI-3	0.41	201	147	472	491	55.3	62
PI-4	0.38	242	221	491	528	60.8	30
PI-5	0.57	310	291	472	519	58.3	26

^a^ Obtained at the baseline shift in the second heating DSC traces.

^b^ Measured by DMA at a heating rate of 5 °C /min.

^c^ 5% weight loss (T5%) and 10% weight loss (T10%) temperatures measured by TGA.

^d^ Residual weight retention at 800 °C.

^e^ CTE, coefficients of thermal expansion measured at a heating rate of 10 °C/min.

**Figure 5.**
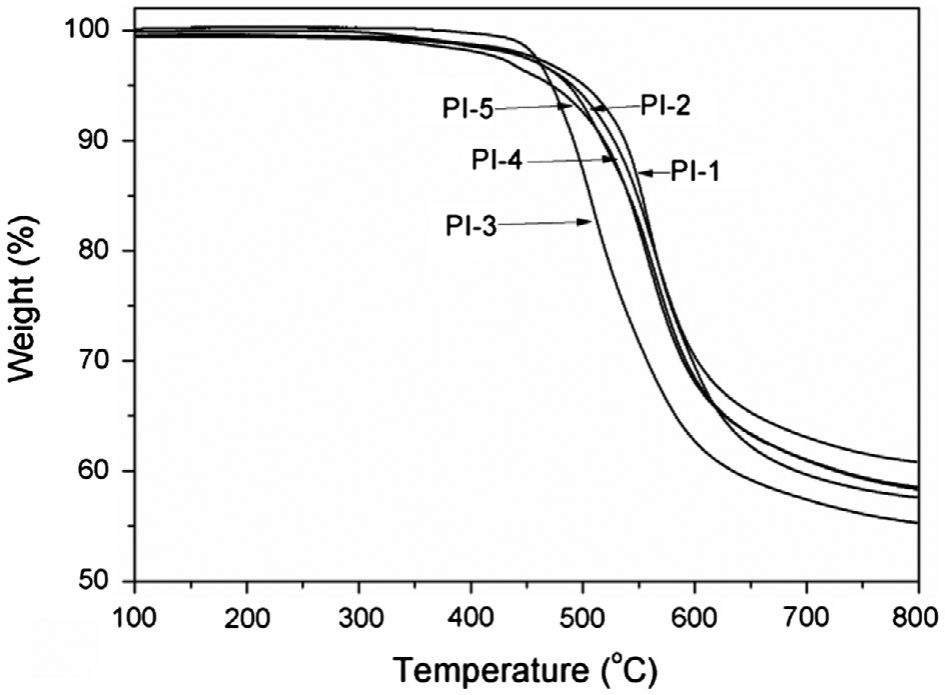
TGA curves of the polyimide films.

**Figure 6.**
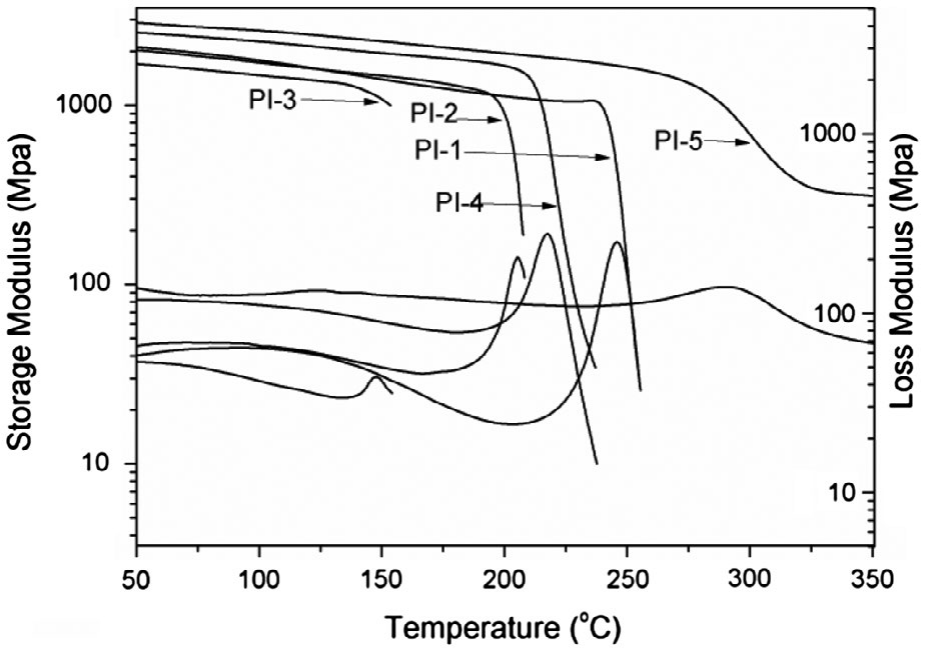
DMA curves of the polyimide films.

The thermal stability properties of the polyimides were obtained by TGA in nitrogen at a heating rate of 10 °C/min, and showed in Table 2. The temperatures for 5%wt loss and 10%wt loss of polyimides in nitrogen atmosphere were obtained from Figure 5 in the range of 472–501 °C and 491–537 °C, respectively. Meanwhile, the char yields at 800 °C were in the range of 55.3–60.8%. From the 10% weight loss temperatures in nitrogen, the following relative order of thermal stability was observed: PI-1 > PI-4 > PI-5 > PI-2 > PI-3. PI-3 having more ether-connecting groups had the lowest decomposition temperature and char yield at 800 °C.

**Figure 7.**
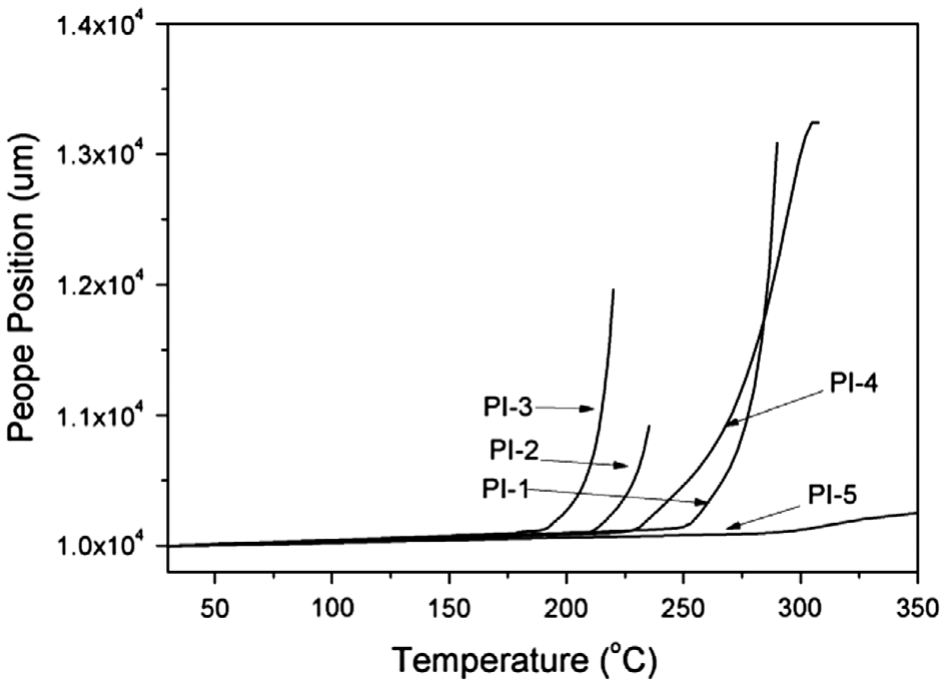
TMA curves of the polyimide films.

As shown in Figure 7, the TMA curves of the polyimide films were obtained at the heating rate of 10 °C/min, and listed in Table 2. The CTEs of the polyimides were among 26–62 ppm °C^−1^. CTEs were influenced by the rigidity and linearity of the polymer chains.[19] The rigidity relative order of dianhydrides was listed as follow: PMDA > BTDA > 6FDA > ODPA > BPADA. All the polyimides were conformed to this feature, and PI-5 possessed the lowest CTE value due to its highest rigidity and linearity of the polymer chain. It is understandable also from no obvious glass transition of PI-5. PI-3 exhibited highest CTE value, because BPADA possesses lowest rigidity and linearity in these dianhydrides.

### 3.4. Mechanical properties of the polyimides

The tensile property of the polyimide films with 25–30 mm thick, 3 mm wide and 4 cm long were measured by a Shimadzu AG-I universal testing apparatus with crosshead speed of 5 mm/min at room temperature, and summarized in Table 3. The films had tensile strengths of 103–145 MPa, an elongation at break of 12.9–15.2%, and a tensile modulus of 1.20–1.88 Gpa, respectively. The mechanical properties of PI-4, 5 were better than that of others, which might be attributed to rigidity structure in the polyimide backbone.

**Table 3.** Mechanical and optical properties of PI films.

Code	*T*_S_ (MPa)^a^	*T*_M_ (GPa)^b^	*E*_B_ (%)^c^	*λ*_cut-off_ (nm)^d^	Transmittance (%)^e^	WU (%)^f^
PI-1	121	1.57	14.5	357	89.7	1.12
PI-2	138	1.66	15.0	372	88.6	1.41
PI-3	103	1.20	15.2	376	86.3	0.37
PI-4	145	1.88	13.1	385	65.8	2.09
PI-5	128	1.87	12.9	391	29.2	1.56

^a^ *T*_s_, Tensile strength.

^b^ *T*_M_, Tensile modulus.

^c^ *E*_B_, Elongation at break.

^d^λ_cut-off_, Cut-off wavelength

^e^ Transmittance at 450 nm.

^f^ Water uptakes (WU).

### 3.5. Solubility and X-ray diffraction of the polyimides

To obtain the solubility of the polyimide films, dissolving 10 mg of polymers in 1 ml of solvent at room temperature for 24 h, and the results were listed in Table 4. The PIs, except PI-3, showed good solvents resistance, which were insoluble in common solvents such as NMP, DMSO, DMAc, DMF, m-cresol. PI-3 exhibited excellent solubility in common solvents owing to the existence of –CH_3_ units and flexible ether linkage of BPADA.

**Table 4.** Solubility behavior of the polyimides containing pyridine.^a^

Solvent	PI-1	PI-2	PI-3	PI-4	PI-5
DMAc	–	–	++	–	–
DMF	–	–	++	–	–
DMSO	–	–	–	–	–
CHCl_3_	–	–	++	–	–
THF	–	–	+-	–	–
Pyridine	–	–	++	–	–
NMP	–	–	++	–	–
Cyclohexanone	–	–	–	–	–
*m*-Cresol	–	–	–	–	–
CH_3_COOH	–	–	–	–	–

Note: ++, soluble at room temperature; +-, partial soluble; –, insoluble.

^a^ Solubility was determined with 10 mg of polyimides in 1 ml of solvent at room temperature for 24 h.

**Figure 8.**
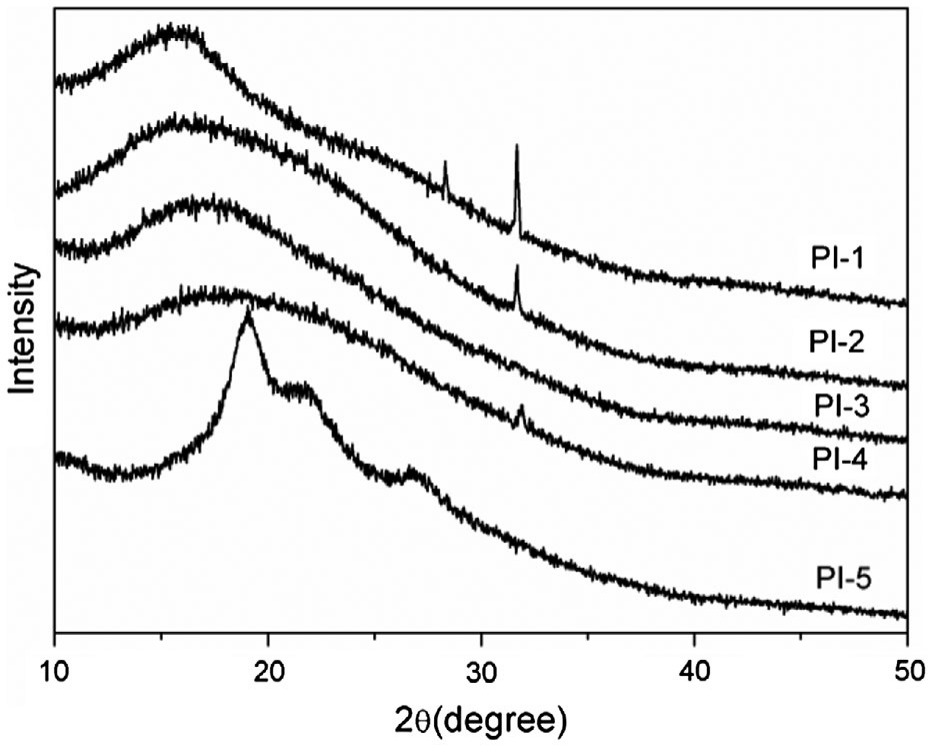
XRD curves of the polyimide films.

Wide-angle X-ray diffraction analysis results showed that the X-ray diffraction curves of polyimides in Figure 8 exhibited almost amorphous patterns apart from PI-5, by which the polyimides were amorphous due to exist the flexible ether linkage loosening the chain packing of the polymer. PI-5 exhibited semi-crystalline pattern because PI-5 derived from PMDA possessed the rigidity polymer chain structure in favor of crystallization.

### 3.6. Optical properties of the polyimides

**Figure 9.**
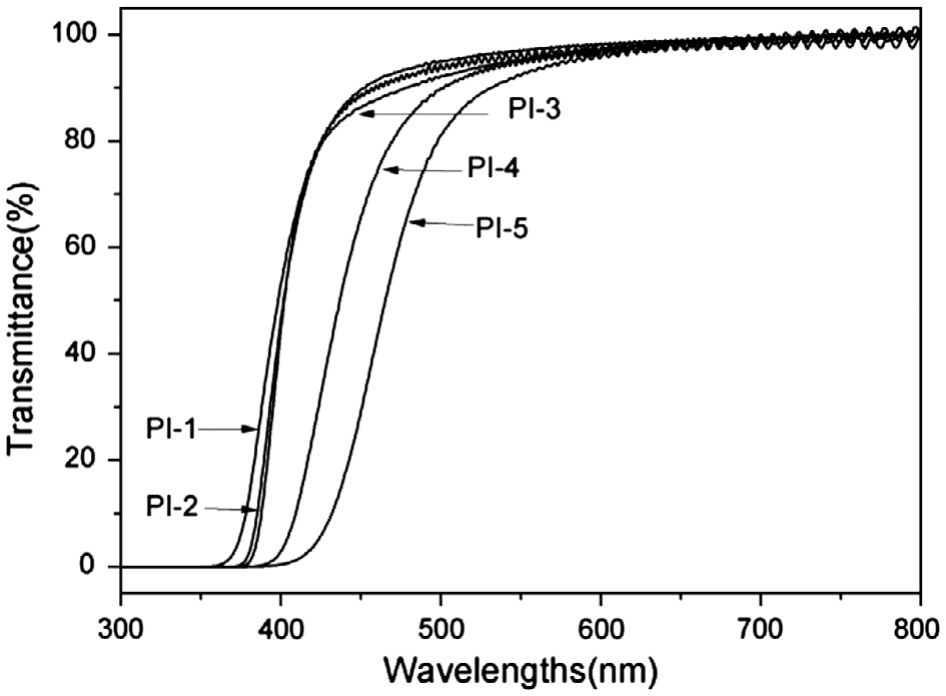
UV–vis spectras of the polyimide films.

The polyimide films with about 30 μm thick as the sample were measured for optical transparency property with UV–vis spectroscopy and the UV–vis spectra were given in Figure 9. The results, percentage transmittances at 450 nm and cut-off wavelengths (*λ*_cut-off_), were listed in Table 3. The cut-off wavelengths (*λ*_cut-off_) were in the range of 357–391 nm, and the percentage transmittances at 450 nm were in the range of 29.2–89.7%. Because the trifluoromethyl groups could inhibit the formation of the CTC （charge-transfer complex） between polymer chains through steric hindrance and the inductive effect, the PI-1 film shows higher transparency at 450 nm and lower *λ*_cut-off_ than the others in Figure 9.[20]

### 3.7. WU of the polyimides

WU of the films were calculated by the following equation: WU = [(*W*_wet_ − *W*_dry_)/*W*_dry_] × 100%; where *W*_wet_ refers to the weight of film samples after immersion in deionized water at room temperature for 24 h, and *W*_dry_ is the initial weight of them. The WU of polyimides were in the range of 0.37–2.09%, and listed in Table 3. Song et al. [21] reports WU of Upilex®-75S (Ube Industries Ltd, Japan) was 1.49% under the same conditions. Compare with it, these results implied that introduction of pyridine and ketone moieties did not deteriorate the water absorption behavior of the PIs. Moisture absorption behavior of polymer could be affected by chemical structure. PI-4 showed highest moisture uptake in comparison to the others owing to the presence of more ketone polar groups which formed hydrogen bonding with H_2_O. PI-3 exhibited the lowest moisture absorption (0.37%), and this result may be attributed to the fact that polyimide contained the water proofing effect of –CH_3_ units and flexible ether linkages of BPADA.

## 4. Conclusions

A new diamine containing pyridine and ketone, BADBP, was designed and prepared through the nucleophilic substitution reaction of 4,4’-dihydroxybenzophenone and 2-chloro-5-nitropyridine followed by the catalytic reduction of the dinitro intermediate with SnCl_2_·2H_2_O and concentrated hydrochloric acid at refluxing temperature. A series of polyimides were obtained from the diamine with commercially aromatic dianhydrides by thermal imidization. The resulting polyimides exhibited excellent thermal stability and *T*_g_s of these polyimides were in the range of 201–310 °C. All the polyimides showed high tensile modulus in the range of 103–145 Mpa. To sum up, this series of polyimides had a good combination of properties applied for high-performance materials and showed promising potential applications in the fields of optoelectronic devices.

## Disclosure statement

No potential conflict of interest was reported by the authors.

## References

[1] Mittal KL Polyimides and other high temperature polymers: synthesis, characterization and applications. Vol. 5 New York (NY): Taylor & Francis; 2009 10.1163/ej.9789004170803.i-424

[2] Liaw DJ, Wang KL, Huang YC, et al Advanced polyimide materials: syntheses, physical properties and applications. Prog. Polym. Sci. 2012;37:907–974. 10.1016/j.progpolymsci.2012.02.005

[3] Liu Y, Xing Y, Zhang Y, et al Novel soluble fluorinated poly(ether imide)s with different pendant groups: synthesis, thermal, dielectric, and optical properties. J. Polym. Sci. A: Polym. Chem. 2010;48:3281–3289. 10.1002/pola.24111

[4] Liu J, Chen G, Fang X Preparation, characterization, and properties of poly(thioether ether imide)s from isomeric bis(chlorophthalimide)s and bis(4-mercaptophenyl) ether. High Perform. Polym. 2015;27:329–337.

[5] Liu J, Nakamura Y, Shibasaki Y, et al High refractive index polyimides derived from 2,7-bis(4-aminophenylenesulfanyl)thianthrene and Aromatic Dianhydrides. Macromolecules. 2007;40:4614–4620. 10.1021/ma070706e

[6] Guan Y, Wang C, Wang D, et al High transparent polyimides containing pyridine and biphenyl units: synthesis, thermal, mechanical, crystal and optical properties. Polymer. 2015;62:1–10. 10.1016/j.polymer.2015.02.009

[7] Song GL, Wang DM, Zhao XG, et al Synthesis and properties of polyimides-containing benzoxazole units in the main chain. High Perform. Polym. 2013;25:354–360. 10.1177/0954008312466278

[8] Song GL, Zhang Y, Wang DM, et al Intermolecular interactions of polyimides containing benzimidazole and benzoxazole moieties. Polymer. 2013;54:2335–2340. 10.1016/j.polymer.2013.02.051

[9] Zeng K, Zou Y Synthesis and properties of polyimides derived from a new phthalonitrile-containing diamine with high polyaddition reactivity. Des. Monomers Polym. 2014;17:186–193. 10.1080/15685551.2013.840500

[10] Dong W, Guan Y, Shang D Novel soluble polyimides containing pyridine and fluorinated units: preparation, characterization, and optical, dielectric properties. RSC Adv. 2016;6:1913–1921.

[11] Zhao J, Peng L, Zhu YL, et al Synthesis and memory characteristics of novel soluble polyimides based on asymmetrical diamines containing carbazole. Polymer. 2016;91:118–127. 10.1016/j.polymer.2016.03.067

[12] Yang Y, Jung Y, Cho MD, et al Transient color changes in oxidative-stable fluorinated polyimide film for flexible display substrates. RSC Adv. 2015;5(71):57339–57345. 10.1039/C5RA06066D

[13] Kumar SV, Yu HC, Choi J, et al Structure–property relationships for partially aliphatic polyimides. J. Polym. Res. 2011;18:1111–1117. 10.1007/s10965-010-9513-2

[14] You NH, Nakamura Y, Suzuki Y, et al Synthesis of highly refractive polyimides derived from 3,6-bis(4-aminophenylenesulfanyl)pyridazine and 4,6-bis(4-aminophenylenesulfanyl)pyrimidine. J. Polym. Sci. A: Polym. Chem. 2009;47:4886–4894. 10.1002/pola.v47:19

[15] Speight JG Lange’s hand book of chemistry. 16th ed New York (NY): McGraw-Hill; 2005.

[16] Chen JC, Wu JA, Chang HW, et al Organosoluble polyimides derived from asymmetric 2-substituted-and 2,2′,6-trisubstituted-4,4′-oxydianilines. Polym. Int. 2014;63:352–362. 10.1002/pi.2014.63.issue-2

[17] Wang JY, Liu C, Su GX, et al Synthesis and characterization of organo-soluble polyimides containing phthalazinone and bicarbazole moieties in the main chain. High Perform. Polym. 2012;24:356–365. 10.1177/0954008312437587

[18] Shang YM, Fan L, Yang SY, et al Synthesis and characterization of novel fluorinated polyimides derived from 4-phenyl-2,6-bis[4-(4′-amino-2′-trifluoromethyl-phenoxy)phenyl]pyridine and dianhydrides. Eur. Polymer J. 2006;42:981–989. 10.1016/j.eurpolymj.2005.11.015

[19] Hasegawa M Semi-aromatic polyimides with low dielectric constant and low CTE. High Perform. Polym. 2001;13:S93–S106. 10.1088/0954-0083/13/2/309

[20] Mulliken RS Molecular compounds and their spectra. II. J. Am. Chem. Soc. 1952;74:811–824. 10.1021/ja01123a067

[21] Song GL, Wang DM, Zhao XG, et al Synthesis and properties of polyimides-containing benzoxazole units in the main chain. High Perform. Polym. 2013;25:354–360. 10.1177/0954008312466278

